# Knowledge Base Commons (KBCommons) v1.1: a universal framework for multi-omics data integration and biological discoveries

**DOI:** 10.1186/s12864-019-6287-8

**Published:** 2019-12-20

**Authors:** Shuai Zeng, Zhen Lyu, Siva Ratna Kumari Narisetti, Dong Xu, Trupti Joshi

**Affiliations:** 1 Department of Electrical Engineering and Computer Science, University of Missouri-Columbia, Columbia, MO USA; 2 Christopher S. Bond Life Sciences Center, University of Missouri-Columbia, Columbia, MO USA; 3 MU Institute for Data Science and Informatics, University of Missouri-Columbia, Columbia, MO USA; 4 Department of Health Management, Informatics University of Missouri-Columbia, Columbia, MO USA

**Keywords:** Knowledge Base, Genomics, Multi-omics data, Organism-specific database, Visualization and analysis

## Abstract

**Background:**

Knowledge Base Commons (KBCommons) v1.1 is a universal and all-inclusive web-based framework providing generic functionalities for storing, sharing, analyzing, exploring, integrating and visualizing multiple organisms’ genomics and integrative omics data. KBCommons is designed and developed to integrate diverse multi-level omics data and to support biological discoveries for all species via a common platform.

**Methods:**

KBCommons has four modules including data storage, data processing, data accessing, and web interface for data management and retrieval. It provides a comprehensive framework for new plant-specific, animal-specific, virus-specific, bacteria-specific or human disease-specific knowledge base (KB) creation, for adding new genome versions and additional multi-omics data to existing KBs, and for exploring existing datasets within current KBs.

**Results:**

KBCommons has an array of tools for data visualization and data analytics such as multiple gene/metabolite search, gene family/Pfam/Panther function annotation search, miRNA/metabolite/trait/SNP search, differential gene expression analysis, and bulk data download capacity. It contains a highly reliable data privilege management system to make users’ data publicly available easily and to share private or pre-publication data with members in their collaborative groups safely and securely. It allows users to conduct data analysis using our in-house developed workflow functionalities that are linked to XSEDE high performance computing resources. Using KBCommons’ intuitive web interface, users can easily retrieve genomic data, multi-omics data and analysis results from workflow according to their requirements and interests.

**Conclusions:**

KBCommons addresses the needs of many diverse research communities to have a comprehensive multi-level OMICS web resource for data retrieval, sharing, analysis and visualization. KBCommons can be publicly accessed through a dedicated link for all organisms at http://kbcommons.org/.

## Background

Large amounts of multi-level ‘OMICS’ data for many organisms have been generated in the recent years due to advancement in next-generation sequencing (NGS) techniques and decreasing sequencing costs. Many genome databases and multi-omics databases have been developed such as MaizeGDB [1], Saccharomyces Genome Database [2], Ensembl genome browser [3], Phytozome [4], GEO [5] and the NCBI BioSystems database [6]. However, genome data and multi-omics datasets are often stored in multiple repositories and usually have many different formats, making integrating them efficiently extremely difficult. Further, multi-omics data analysis tools and visualization tools are not available in these databases. To address this, we have designed and implemented Soybean Knowledge Base [7, 8] (SoyKB), a one-stop shop web-based resource for soybean translational genomics research. It plays a role in central data repository aggregating soybean multi-omics data, and contains various bioinformatics tools for data analysis and visualization. It is publicly available at http://soykb.org, and has wide range of usage around the world, with more than 500 registered users. For newly studied and discovered organisms with no existing databases, users interested in other organisms such as viruses, microbes, biomedical diseases, animals and plants also have very similar needs. Thus, a centralized repository to address such needs is necessary. There is also a growing need to tap into genomics findings from other model plants and animals by conducting cross-species comparative analyses. Researchers working on multiple organisms and interested in comparing datasets from different species, would otherwise have to spend their valuable time in familiarizing themselves with different databases and their layouts. Without a comprehensive centralized database system, it generally consumes a lot of time with a repetitive and manual procedure of extracting and organizing all information one by one. Providing a comprehensive and flexible framework which are more customized and developed to support cross-species translational research is a need.

To achieve this, we have designed and developed KBCommons [9] v1.1, which is an all-inclusive framework supporting genome data and multi-omics dataset retrieval, multi-omics data analysis and visualization, and new organism database updating and creation. It provides six entities information including genes/proteins, SNP, microRNAs/sRNAs, traits, metabolites as well as animal strains / plant germplasms / patient populations / viral or bacterial strains, etc. Several multi-omics datasets including phenomics, epigenomics, genomics, transcriptomics, proteomics, metabolomics and other types are also incorporated in KBCommons. The KBCommons v1.1 framework and tools are currently supporting *Zea mays*, *Arabidopsis thaliana*, *Mus musculus*, *Homo sapiens, Rattus norvegicus, Canis familiaris* and *Caenorhabditis elegans* KBs. It provides a suite of tools such as the Heatmaps, Hierarchical Clustering, Scatter Plots, Pathway Viewer and Multiple Gene/Metabolite Viewer. It also provides interface to access to PGen [10] and Pegasus Analytics Workflows for genomics variations analysis and for newly developed RNAseq workflows respectively. To visualize differential expression analysis in transcriptomics dataset, KBCommons provides a suite of visualization tools including Venn Diagrams, Volcano Plots, Function Enrichment and Gene Modules. A functionalities of data sharing and data releasing are contained in it. Without having to reinvent the wheel for every organism individually, using KBCommons to expand our background framework, in-house visualization and analysis tools from SoyKB to other organisms, provides a ready-to-use and efficient option for users from all biological domains and reduces the time in development significantly. The similar layout for information access across organisms is provided in each KBs making it easier to users to utilize data from across multiple species and navigate through the system.

## Methods

**Fig. 1:**
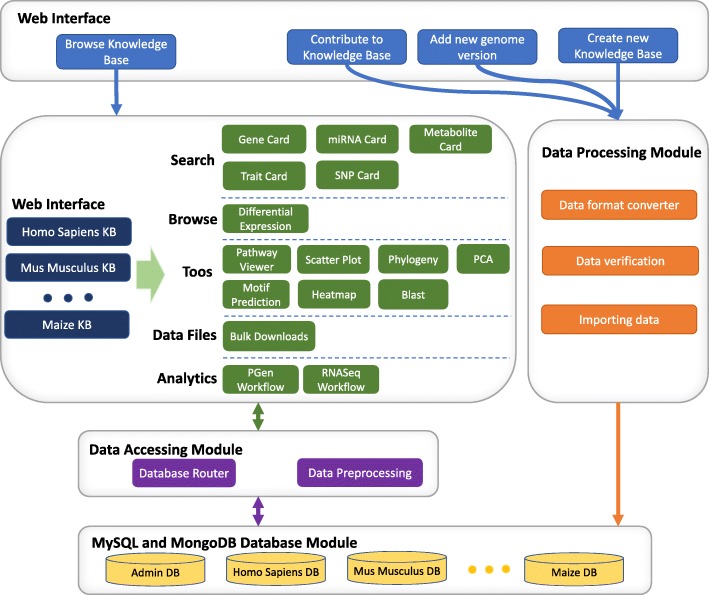
KBCommons framework. KBCommons architecture showing the database, data processing, data accessing and web interface modules

The KBCommons v1.1 framework is maintained on the CyVerse [11, 12] advanced computing infrastructure. KBCommons utilizes the Extreme Science and Engineering Discovery Environment [13] (XSEDE) and CyVerse data store cloud storage to access analyzed datasets to load them into the tools directly and store raw datasets and perform data analysis. KBCommons v1.1 is hosted on Apache [14] server and implemented using the Laravel [15] PHP web framework. KBCommons is designed to be user-friendly and using HTML, JavaScript [16], AngularJS [17], and Bootstrap [18] in the front-end. To visualize data interactively, the Highcharts [19] and Google Charts [20] are used in KBCommons. The architecture of KBCommons composes of four modules which are shown in Fig. 1 and details are described below.

### MySQL and MongoDB database module

We utilize two types of databases, MySQL [21] and MongoDB [22], to manage biological data including genomic data, multi-omics experimental data, functional annotation data, and other associated users profile and groups information. The database module integrates various genomic data and multi-omics data including phenomics, epigenomics, genomics, transcriptomics, proteomics, metabolomics, annotated whole genome sequences, etc. for many organisms. The database module also incorporates the authentication and authorization information for public vs. private datasets and permissions established by users for data sharing.

### Data processing module

This module is connecting KBCommons interface module and database module by processing users uploaded genomic and multi-omics data, and by importing those data. It developed using Python [23] and Python based high-performance data analysis package named Pandas [24]. The module composes of a series of efficient pipelines from data verification to data imputation, which are fully automated and require no manual processing steps in between. Using this module, users can upload new gene models, genome sequences and annotations features downloaded from Ensembl [25] or Phytozome to create a new KB. Phytozome is the preferred suggested data source for plant species, while Ensembl for all non-plant species for standardized formats for genome sequence and annotations datasets. The results of multi-omics datasets analysis such as results from RNAseq analysis tools such as Cufflink [26], Cuffdiff [26], Voom [27] and EdgeR [28] can also be uploaded via this module.

### Data accessing module

This module is a data retrieval component to accesses data according to users’ keyword searching, type of dataset, functionality of tools. It is implemented in PHP [29], which is a popular programming language originally designed for web development. To access the same type of experimental data for different organism database without duplicating the code, it accesses database dynamically by a given experimental data conditions and its response of routing strategy. It has an array of general and shareable data processing sub-modules to avoid over-engineering.

### Web Interface module

This module uses JavaScript-based interactive charts libraries, the Highcharts and Google Charts to visualize data interactively. It is designed and developed to provide easy access to user’s experimental data based on searched conditions. It allows users to create groups and set up proper permission of data for data sharing. The Hierarchical design is applied to the front-end display to not only facilitate user access to the most interesting portions of the database but also to provide a comprehensive view to explore the data from all aspects.

## Results

### KBCommons accounts, groups and data sharing

#### Account registration

KBCommons allows users to create personal account in the sign-up page with required information. Users can modify their personal profile, upload profile picture, and list all groups in KBCommons once they have completed the registration. With their accounts, users can bring in their private dataset for any organism and visualize any public or sharable dataset via KBCommons interface.

#### Creation of groups

Creating collaborative groups options are available for all users. The groups’ creators have all privileges to approve or reject any requests to join their group. All requests to join a group would be sent via KBCommons notification system. The creators of groups also have privileges to manage datasets, to share datasets with group members or to delete datasets. All groups are listed along with details of groups and status of the request in users’ profile page.

#### Sharing data with group members

All uploaded datasets are private by default and their ownership and access permissions can be modified by owner. Owner of dataset can share dataset to any groups and group members with their dataset privilege. All of group members having access permission can retrieve and visualize shared data.

### KBCommons key features

#### Creating a new Knowledge Base

**Fig. 2:**
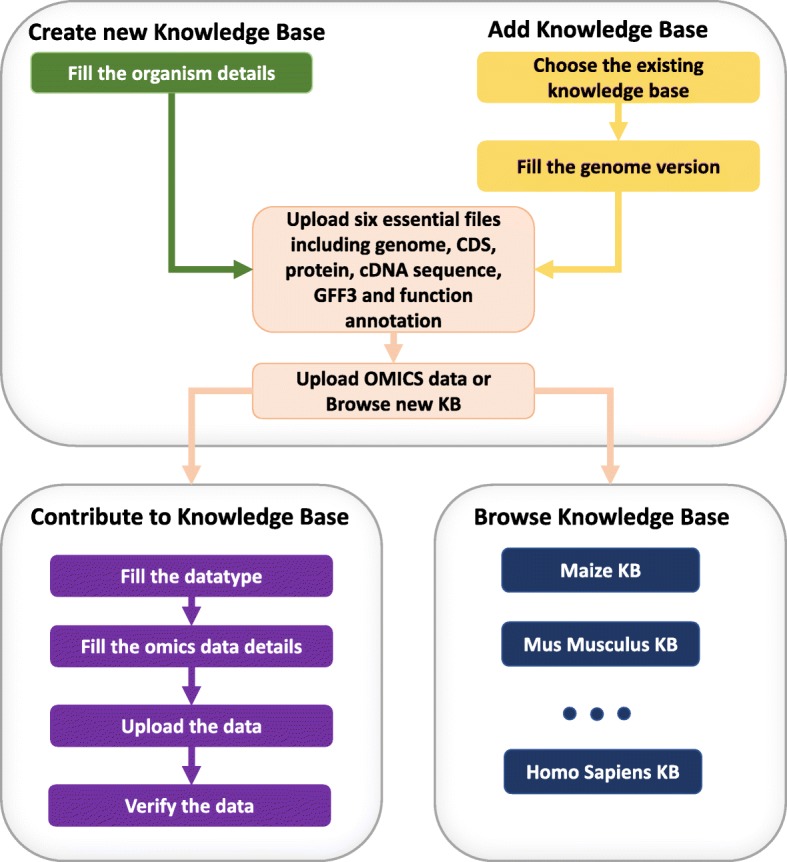
Workflow of creating Knowledge Base and data contribution. The workflow showing processing of the creation and contribution with essential genome data and OMICS data

KBCommons provides the capacity to import new organism data to KBCommons and create an entirely new KB for organisms not in KBCommons. It also provides an easy-to-use automated procedure to import the 6 essential files including genome, CDS, protein, cDNA sequences, gene annotation and GFF files from Ensembl or Phytozome for animals and plants respectively to our database. Genome version verification is performed after uploading 6 essential files completed by comparing the MD5 checksum for uploaded files and Ensembl or Phytozome original files. The workflow creation of KBs and workflow of data contribution are shown in Fig. 2.

#### Contribution to KBCommons

KBCommons supports uploading users’ new multi-omics data including SNP, Indels, methylation, metabolomics expression, proteomics, RNAseq and microarray, etc. Users can use this feature on any existing KBs or following the creation of new KB for an organism. With data processing module, KBCommons processes uploaded data and imports these data to an appropriate database according to genome version, type of dataset and other customized options. KBCommons supports various standard file formats only including Fasta format for sequences data, FPKM or read count data for gene expression, and VCF format for single nucleotide polymorphisms (SNPs) data to ensure no incorrect or false-positive data is uploaded by user. It also uses validation rule for screening insertion or submission of any junk data / characteristics and incorrect information to prevent invalid data.

#### Adding version to KBCommons

KBCommons allows users to add new genome versions to existing organism KBs and update current organism KBs by uploading the 6 essential files and filling out the organism details such as organism type, name, model version and genome version. KBCommons also uses the data processing module to prepare the required database for further searches and utilization in tools like multiple sequence similarity analysis. Once a user adds a new genome version to existing KB it also enables them to start bringing in multi-omics datasets corresponding to this newly added genome version.

#### KBCommons browsing

In browse KBCommons tab, all of existing organism KBs with their versions are displayed. All of organisms are listed into four main categories including Animals and Pets; Plants and Crops; Microbes and Viruses; Humans and Diseases. Along with this classification, we also provide a model organism section, which displays model organisms from all the categories. All available genome versions are shows as a list in corresponding organisms KB drop down menu.

### Data sources

The data in KBCommons comes from multiple sources. Many of the data incorporated in KBCommons are public data and accessible to all users without login. KBCommons also incorporates and integrates many of private data collected from our collaborators, only available for group members. All of data information are shown in Data Source page in KBCommons home page on the top menu bar. Currently, KBCommons incorporates genome data for *Zea mays, Arabidopsis thaliana, Mus musculus, Homo sapiens, Rattus norvegicus, Canis familiaris* and *Caenorhabditis elegans*. KBCommons also have information about traits, SNPs, annotated metabolites, miRNAs and gene entities. The gene models, genomic sequences and functional annotation information were acquired from Ensembl and Phytozome. KBCommons has experimental data for Illumina RNA-Seq experiments covering various tissue types. KBCommons also hosts data regarding miRNAs and their expression abundances came from Cancer Cell Line Encyclopedia (CCLE) [30] and The Cancer Genome Altas (TCGA) [31] and the microRNA database [32] (miRBase). It also hosts gene expression data of 9264 tumor samples across 24 cancer types came from TCGA. The pathway information is acquired from Kyoto Encyclopedia of Genes and Genomes (KEGG) [33].

### KBCommons search options

**Fig. 3:**
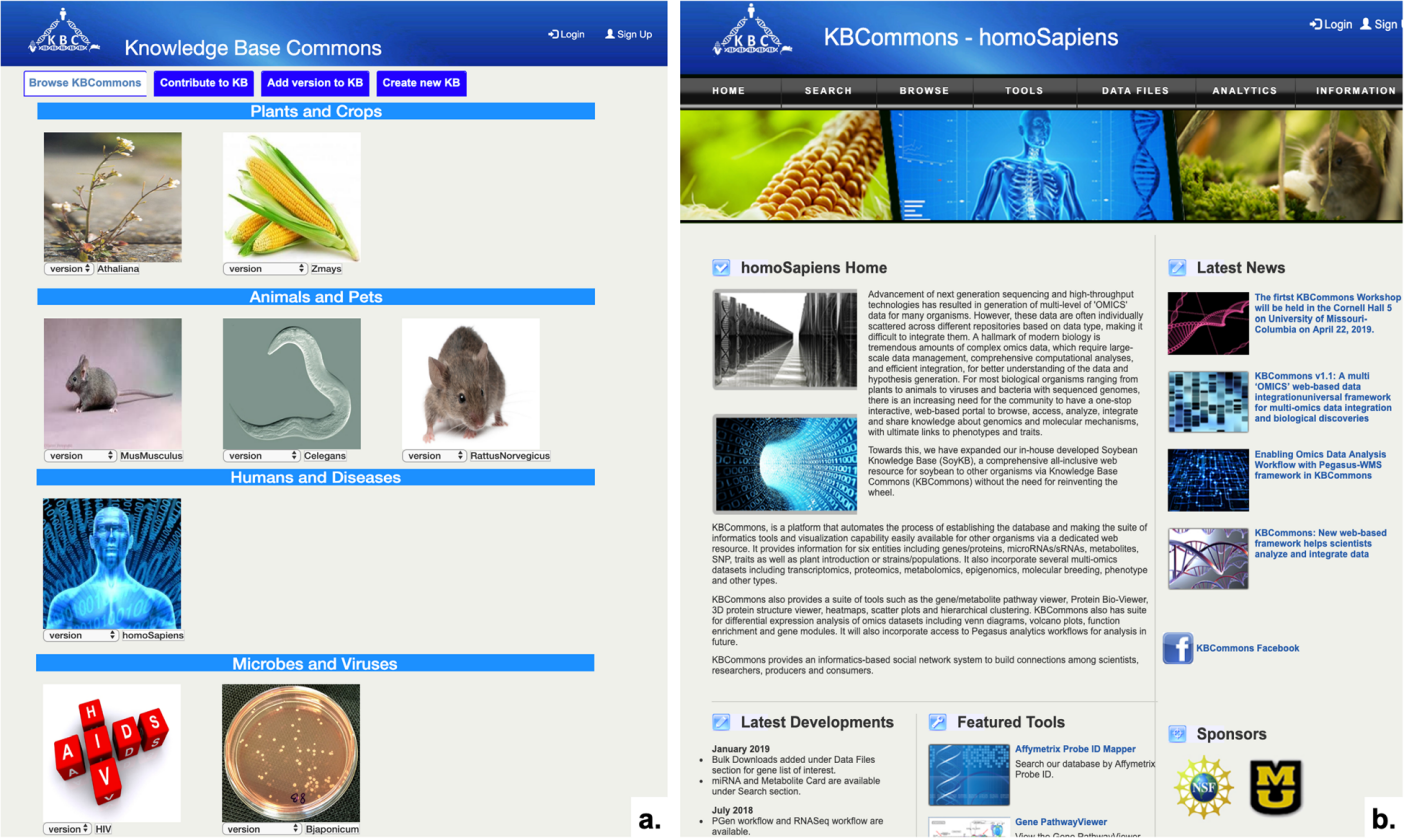
KBCommons home page. **a** KBCommons home page shows Plants and Crops, Animals and Pets, and human and diseases model and corresponding Knowledge Base; **b** Knowledge Base page shows menu bar for navigation, login, and highlight of the developments

The KBCommons home page (Fig. 3a) provides users with entry points to access all features provided by our Knowledge Base. All of Knowledge Base web pages (Fig. 3b) have similar layout and navigation bar at the top for easy access. The navigation bar has links to different sections including Search, Browse, Tools and General Information.

#### Gene card

**Fig. 4:**
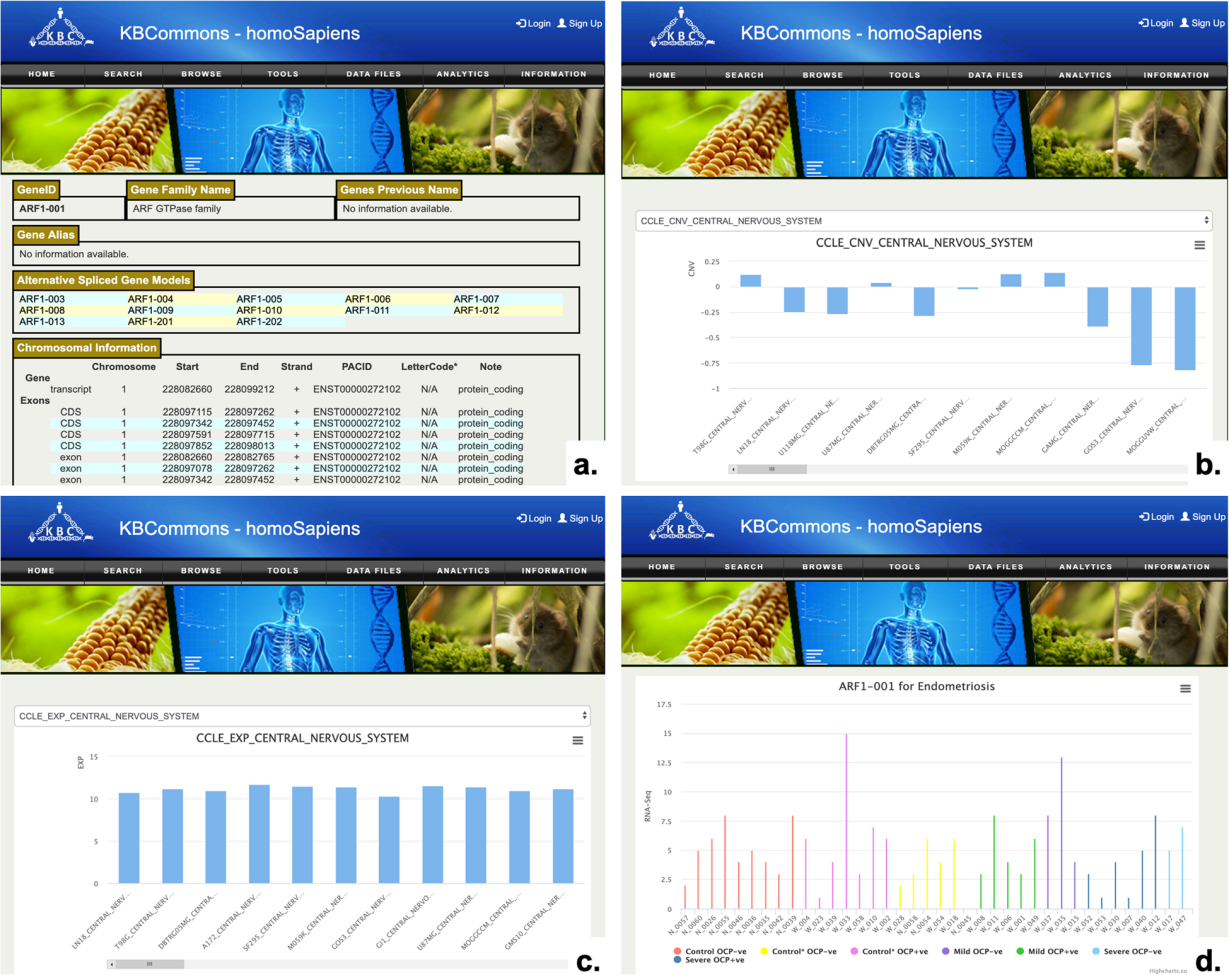
Gene Card. **a** Example of Gene Card page in *Homo sapiens* KB for ARF1–001 shows gene module, gene family name, chromosomal information, function annotation, and corresponding CCLE profiles; **b** Copy Number Variation profiles; **c** Microarray profiles; **d** RNASeq Read Count

The Gene Card page (Fig. 4a) provides users with information about gene name, gene version, gene family, alias names, gene models with the intron, exon, UTRs, chromosomal information including gene coordinates, strand, cDNA, CDS, protein sequences, and functional annotations including Pfam [34] and Panther [35], and links to pathway viewer. It provides visualization tools to show copy number variation (Fig. 4b) data, transcriptomics data from microarray (Fig. 4c) or RNAseq experiments (Fig. 4d), and other omics data types in graphic charts.

#### miRNA card

**Fig. 5:**
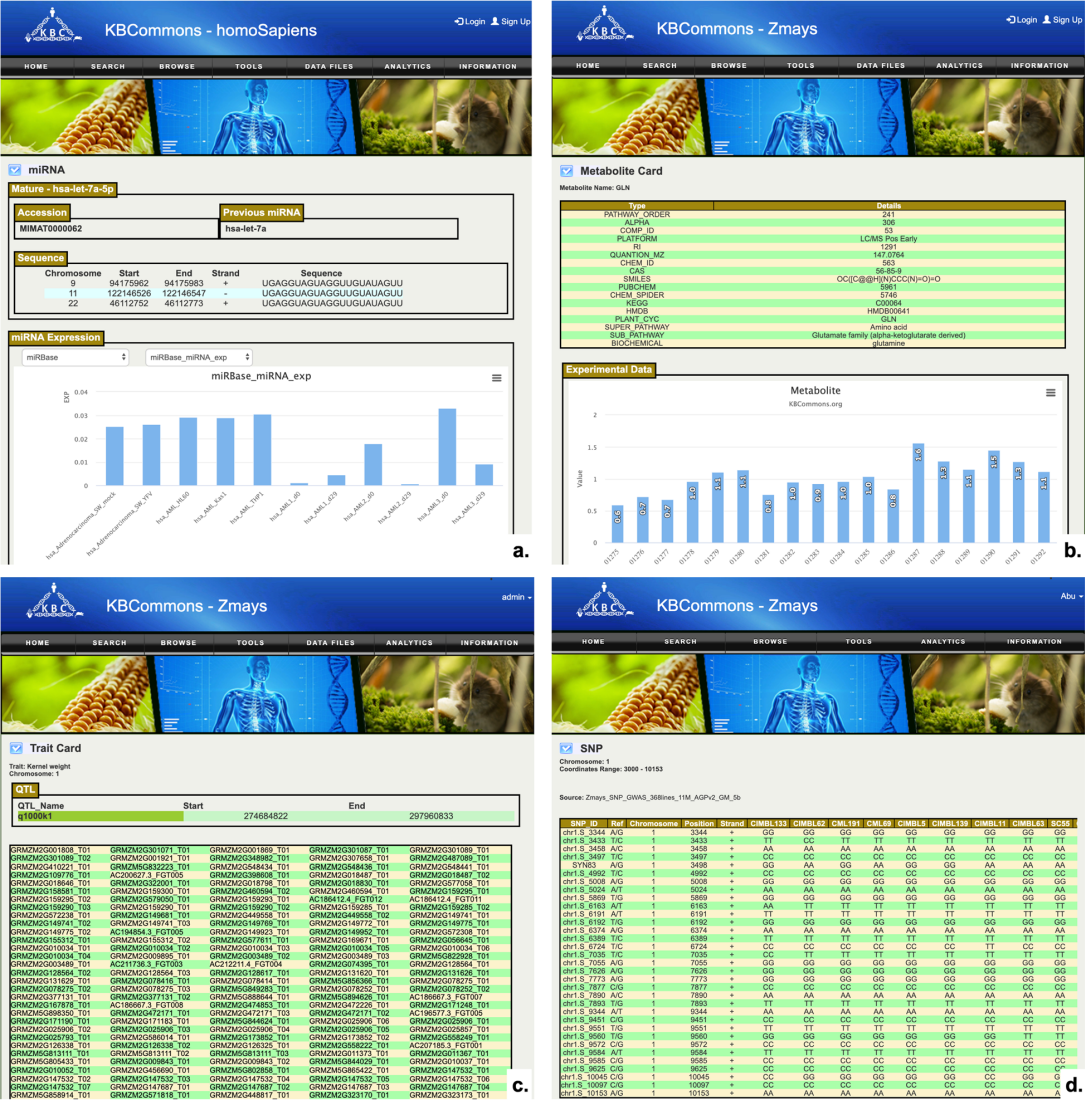
Examples of miRNA, metabolite, Trait and SNP Card. KBCommons provides various ways to access (**a**) miRNA; **b** Metabolite; **c** Trait and (**d**) SNP

The miRNA Card (Fig. 5a) contains information about experimentally validated or predicted miRNAs, mature miRNA sequence, accession ID, and predicted target genes including corresponding gene coordinates, conservation value, align score, binding energy, and mirSVR score. The miRNA expression data from TCGA and miRBase have been incorporated for browsing on miRNA Card pages.

#### Metabolite card

The Metabolite Card (Fig. 5b) stores information about metabolites including alias names, pathway, molecular weight, chemical structure, chemical formula, mass-to-charge ratios and SMILES [36] formula. The expression of metabolomics is plotted as bar chart for easy understanding.

#### Trait card

The Trait Card (Fig. 5c) pages contains information about trait name, multiple QTL regions identified on each of chromosomes, and genes overlapping in individual QTL regions. Information about SNPs, insertions and deletions are also shown in tables.

#### SNP card

In the SNP Card (Fig. 5d), the predicted SNPs, reference bases, their chromosomal positions, and consensus bases are shown in table. The QTL traits and genes where the SNP falls and overlaps within a gene model’s coordinates are also listed.

### KBCommons browse options

#### Differential expression

**Fig. 6:**
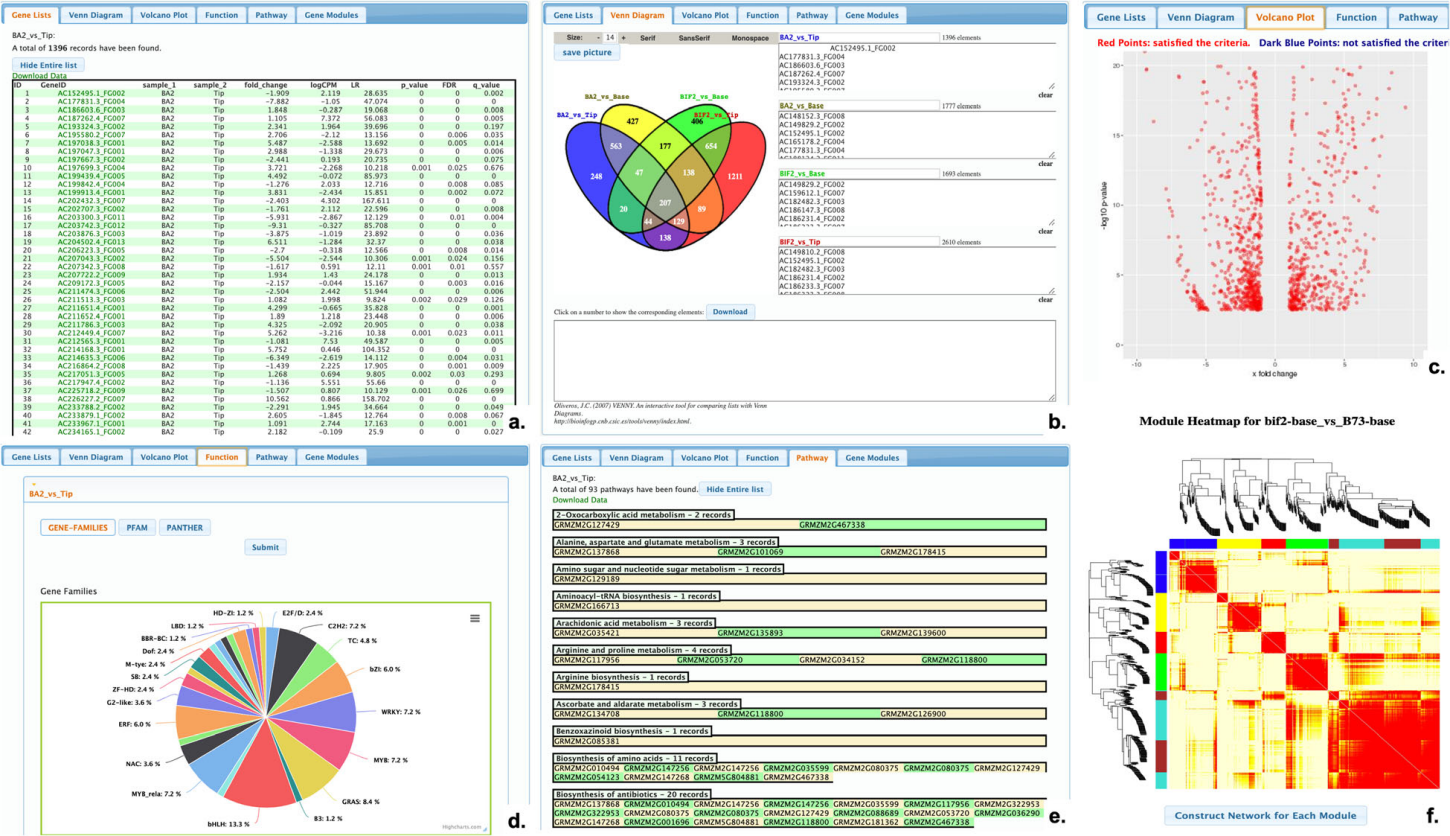
Differential Expression. In Maize KB, the differential expression page for public dataset shows the following: (**a**) Gene lists; **b** Venn diagram; **c** Volcanoplot; **d** Function; **e** Pathway; **f** Gene modules

The Differential Expression provides a set of visualization tools showing the comparison results of transcriptomics data from Cuffdiff [26], VOOM [27] and edgeR [28]. These results can be filtered by *p*-value, q-value, fold change and gene regulation types including down-regulated, up-regulated and both. The Differential Expression have six different tags for Gene Lists, Venn Diagram, Volcano Plot, Function Analysis, Pathway Analysis and Gene Modules. The Gene Lists tab (Fig. 6a) shows a list of genes along with p-value, fold change and links to Gene Page in the form of tables. The Venn Diagram tab (Fig. 6b) visualizes overlapping of differential expression genes in different experimental conditions, and allows users to list and download all of genes name in the overlapping set. In Volcano Plot (Fig. 6c), down-regulated genes or up-regulated gene with log fold change and q-value are shown in scatter charts. In the Function Analysis tab (Fig. 6d), distribution of transcription factor gene families and distribution of protein families are shown as pie charts, and all gene families along with percentage are listed. In the Pathway Analysis tab (Fig. 6e), KEGG athways are categorized, and genes are listed under proper pathway. The Gene Modules (Fig. 6f) shows the correlation patterns among genes expression data identified by weighted correlation network analysis (WGCNA) [37]. All of gene names under these six tabs are linked to appropriate Gene Card pages to retrieve information of gene easily.

### KBCommons tools options

#### Pathway viewer

**Fig. 7:**
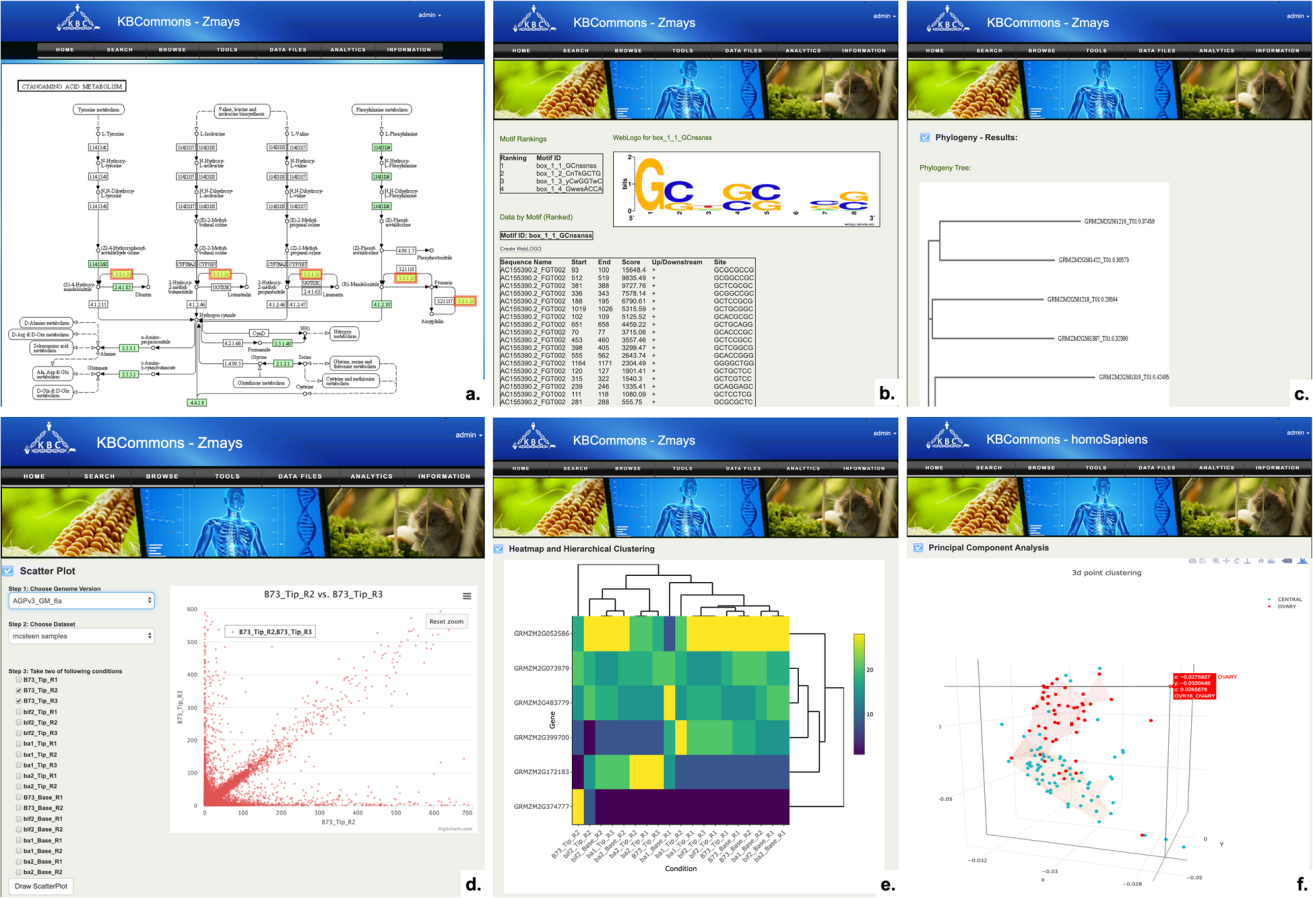
KBCommons Tools. KBCommons has various data analysis and visualization tool including (**a**) Pathway Viewer; **b** Motif Prediction and Web Logo; **c** Phylogeny; **d** Scatter Plot; **e** Heatmap and Hierarchical Clustering tool; **f** Principal Component Analysis

The Pathway Viewer (Fig. 7a) shows KEGG pathways according to list of genes or list of metabolites. The Pathway Viewer provides two ways to show pathway, which are viewing a pathway containing specific compounds/genes and viewing an existing pathway. Downloading pathways mapped for genes and genes mapped for pathway are available.

#### Motif prediction and web logo

The Motif Sampler [38, 39] tool (Fig. 7b) is designed to make generation of web logo of sequence easy and predicts motifs, indicating domains or conserved consensus sequences, on multiple protein or genes sequences. The predicted motifs and ranking score are shown in form of tables. These motifs are visualized in web logo, a graphical representation of nucleic acid multiple sequence or an amino acid alignment.

#### Sequence similarity and phylogeny

The BLAST [40] tool and ClustalW2 [41] are included in KBCommons for pairwise sequences search and for multiple sequences search respectively. These two tools consider the customized parameters, and sequences information such as genome, CDS, cDNA and protein as input. The result of BLAST shows a list of hits starts with the best match and expected number of chance alignments in the Result page. The Phylogeny Tool (Fig. 7c) generates a diagram tree that represents evolutionary relationships among multiple sequences by either neighbor-joining (NJ) [42] method or unweighted pair group method with arithmetic mean (UPGMA) method [43].

#### Scatter plot

The Scatter Plot tool (Fig. 7d) retrieves all available expression datasets and corresponding experimental conditions/replicates. Then it visualizes correlation of genes from two chosen experimental conditions on a scatter chart. The data in scatter plot deviating away from the diagonal represents genes having variations in their expression patterns and it can be detected easily. In the scatter chart, moving cursor over a data point can display its particular expression value.

#### Heatmap and hierarchical clustering

The Heatmap and Hierarchical Clustering tool (Fig. 7e) displays a heat map representing level of expression of genes across multiple experimental conditions. It allows users to enter a list of gene names and experimental conditions to create a heat map. These genes are clustered according to their expression values in different experimental conditions. In the heat map, option to save heat map as an image is available. The operations of zoom in and zoom out are also available by either clicking the zoom in/out button or selecting a region of interest in the heat map.

#### Principal component analysis (PCA)

The PCA tool (Fig. 7f) is used for clustering and visualizing samples grouped by the cancer cell line type by reducing the dimensionality of the multi-dimensional gene expression data to three-dimensions. It projects the whole set or subset of gene expression data chosen by user onto three principle components which can be viewed as a gene-like pattern of expression across the samples. The PCA plots implemented by using Plotly [44] which generated a 3D point clustering chart. The coordinates represent the first three principal components that have the largest possible variance and highlight the most similar and different cancer cell lines based on their closeness and distance.

#### Data analytics

**Fig. 8:**
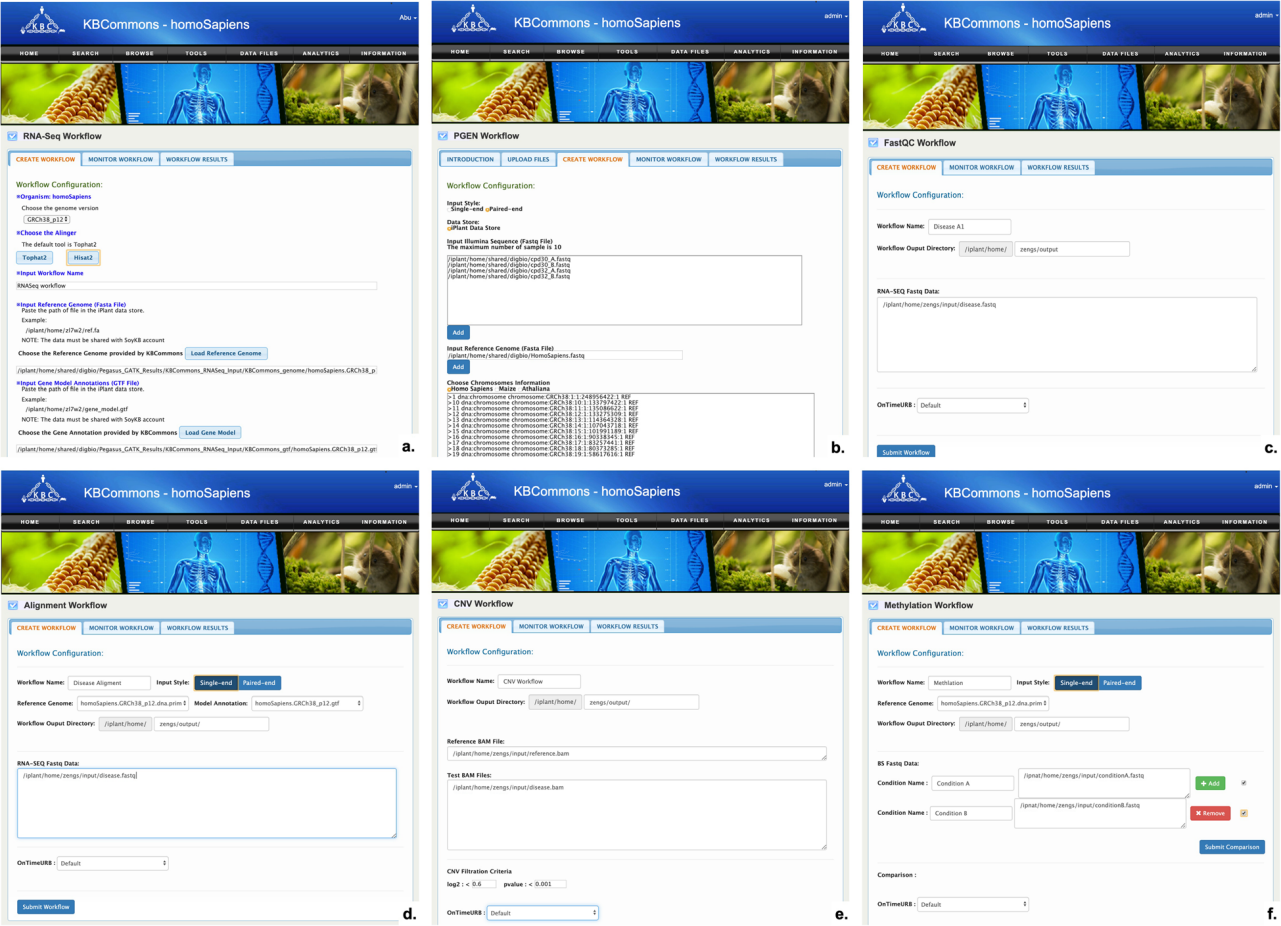
Workflow in KBCommons. KBCommons provides (**a**) RNASeq Workflow for calculating abundance of the transcripts and computing the difference in expression, **b** PGen workflow for SNP and INDELS calling, **c** FastQC Quality Check workflow, **d** Alignment workflow, **e** Copy Number Variation workflow, and (**f**) Methylation workflow

We have implemented two high-throughput cloud-based bioinformatics data analysis workflows in KBCommons: RNA-Seq analysis workflow (Fig. 8a), PGen [10] workflow (Fig. 8b), FastQC Quality Check workflow (Fig. 8c), Alignment workflow (Fig. 8d), Copy Number Variation (CNVs) workflow (Fig. 8e) and Methylation workflow (Fig. 8f). We make all the bioinformatics workflows managed by Pegasus Workflow Management System (WMS) [45] and run them on the XSEDE [13] HPC resources using SoyKB and KBCommons Gateway Analytics allocations.

The RNA-seq analysis workflow is used for performing quantitation of gene expression from RNA-Seq transcriptomics data and statistical analysis to discover differential expressed genes/isoform between various experimental groups/conditions.

The PGen workflow allows users to identify SNPs and insertion-deletions (indels), perform SNP annotations and conduct copy number variations analyses on multiple resequencing datasets in a user-friendly and seamless way.

The FastQC workflow is used to conduct quality control checks on raw NGS data coming from high-throughput sequencing projects, to ensure the data looks good and there are no problems or biases which may affect its further downstream analysis and use.

The Alignment workflow is used to align NGS data or RNA-Seq reads to reference genome. The outputs are in ‘BAM’ format files.

The Copy Number Variation workflow is used to perform efficient analysis to detect CNVs in the form of gains and losses, from NGS reads. This workflow requires user to input a reference sequence and one or more multiple sample/condition sequences which should in ‘BAM’ format. The methylation workflow is used to analysis the high-throughput NGS bisulfite sequencing reads to estimate the methylation level for every cytosine site. There are many other methylation analyses such as hypo-methylated regions (HMRs), hyper-methylated regions (HyperMR) and differentially methylated regions (DMR) between two methylomes can be achieved by this workflow.

#### Data download

**Fig. 9:**
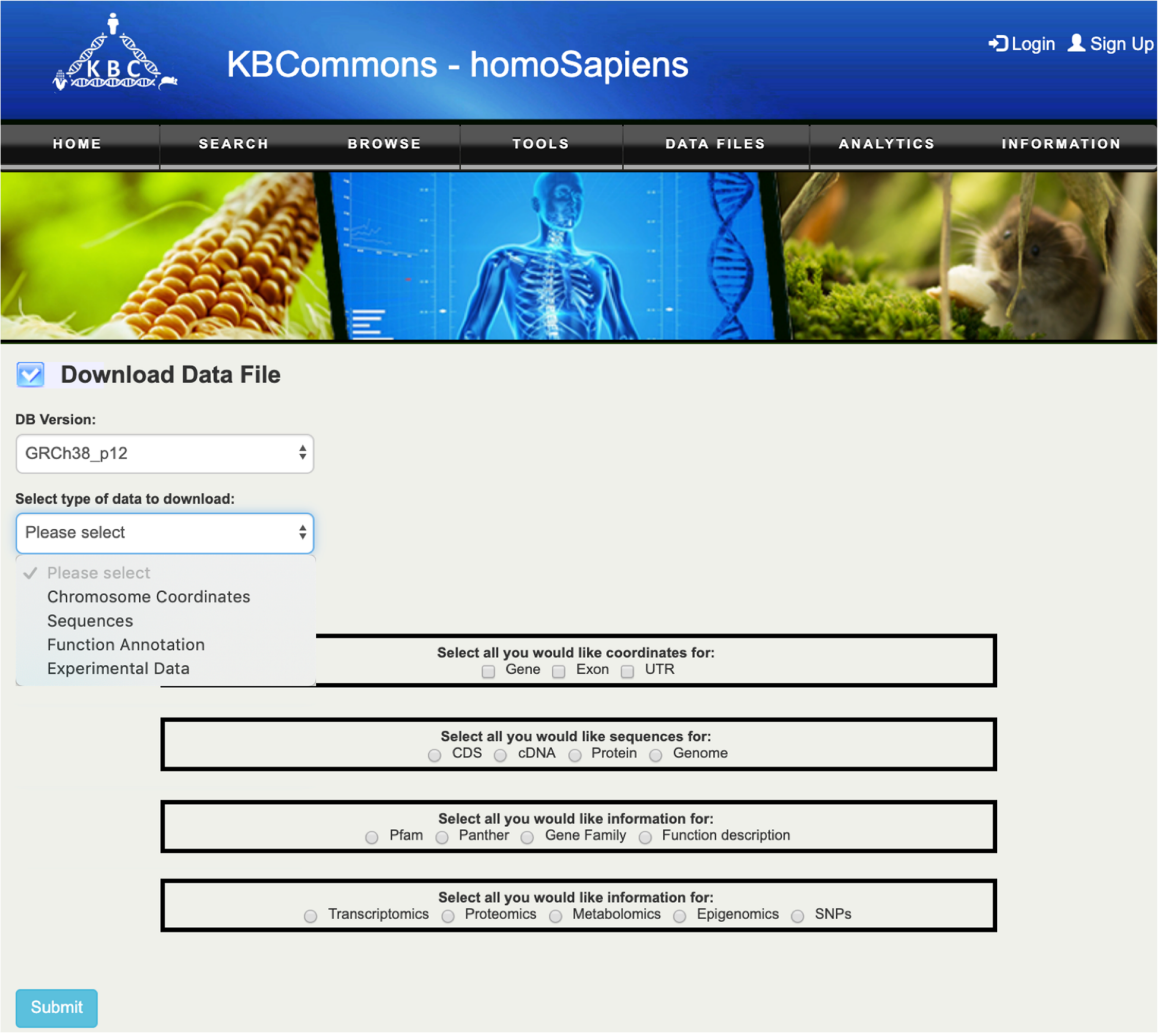
Data Downloads. Bulk Downloads page provides options for downloading domain information, sequences information, chromosome coordinates, and experimental data

The Data Download (Fig. 9) capacity provides an easy access way to allow users to download data for their gene list of interest. Users can choose genome version and type of data for their gene list. The chromosome coordinates for genes, exons and UTR; CDS, cDNA and protein sequences; Pfam, Panther, Gene Family and Function description; are the data currently available for bulk download.

## Use cases for KBCommons application

### Create new Knowledge Base

**Fig. 10:**
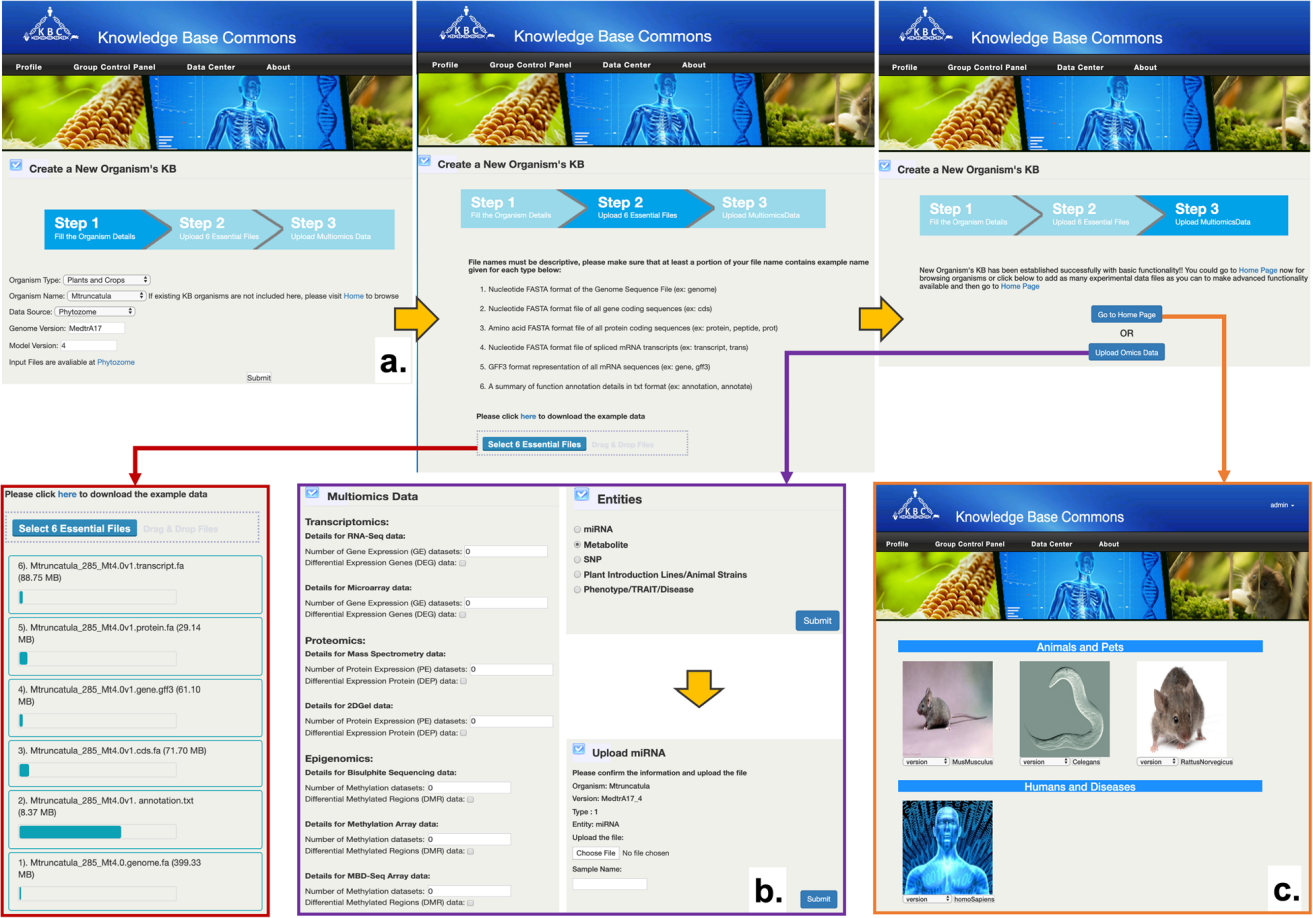
Example of Knowledge Base creation and data contribution. **a** Create new Knowledge Base or add new version to KBs; **b** Data contribution; **c** KBCommons home page

In this section, we show a functionality of new KB creation by importing the genome sequences and general features. We studied *Medicago truncatula,* which is a model organism for legume biology, and is categorized into Plants and Crops type. An example of creating new KB with *Medicago truncatula*’s genome data is shown in Fig. 10. To keep track of owner information who uploads the data and whether data needs to be public or private, registration and login are required before creating a new KB. We firstly access page of Create New Organism’s KB and then populate information about organism name, organism type, genome version and model version. We upload 6 essential files (Fig. 10a-b) including genome sequence, CDS, protein sequences, cDNA and genomic features file downloaded from Phytozome or Ensembl. We can click on the monitor button to show uploading progress in progress bar. We can directly enter the *Medicago truncatula* KB or upload others related multi-omics data to that KB.

### Contribute to MaizeKB and retrieve data

With differential expression dataset generated by Cuffdiff, we show an example of contribution of multiomics data to existing KB. The organism name and genome version we use are *Zmays* and AGPv3_GM_6a respectively. To describe dataset information (Fig. 10b), we then check on the Differential Expression Genes option and enter number of dataset. We further upload a differential expression dataset and submit a task to import dataset to database. Users can retrieve the differential expression data in Differential Expression tools after the task is done.

### Accessing DiseaseKBs

In KBCommons *Homo sapiens* species, we have also developed the capacity for large-scale genomics studies portal to analyses and visualize the data and/or relevant patient de-identified data. Most of the datasets comes from TCGA [31]. The data types from 9000+ tumor samples, 24 cancer type include FPKM and Feature Counts for gene expression as well as clinical parameters. The Endometriosis data include RNAseq read counts and methylation read count by regions for 80 samples covering 7 sub-groups of patients.

#### CancerKB data

**Fig. 11:**
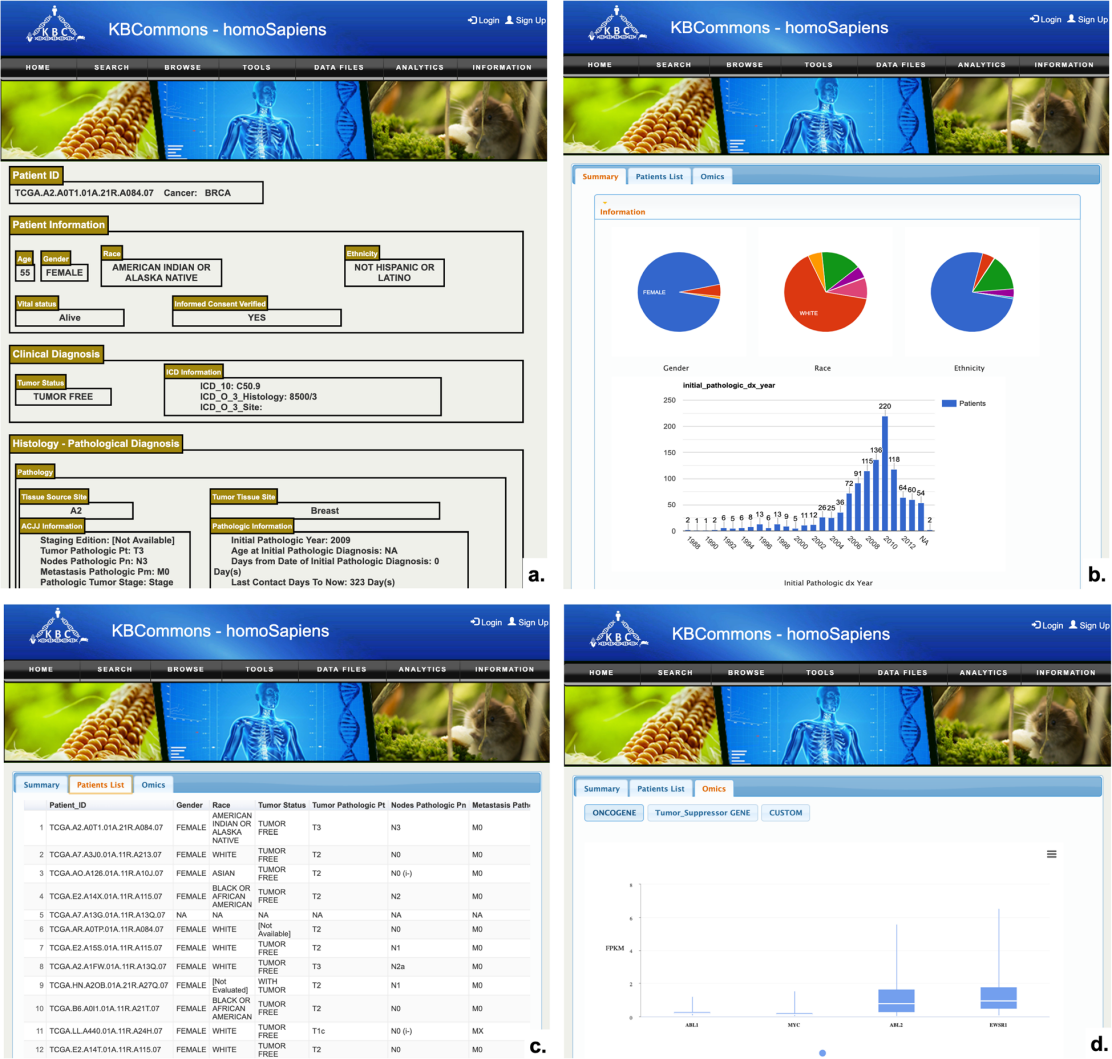
Example of CancerKB Data. **a** Patient page shows patient information including age, gender, cancer status, and corresponding histology information; **b** Summary page shows distribution of patients in gender, race, etc.; **c** Patient List shows all patients information in the form of table; **d** Omics view presents a boxplot of distribution of FPKM in different cancer stages

Users can explore and visualize the patients’ genomics data as well as the de-identified information by queried the TCGA barcode. When available, relative patient information including age, gender, etc.; ICD information; ACJJ information; transcriptomics evidence including the FPKM and Feature Counts charts are shown on the patient page (Fig. 11a). When viewing expression data, there are two pre-defined gene set for options: oncogenes and tumor suppressor genes or user can manually enter gene symbols of interest. Users can select specific gene sets such as oncogenes, tumor suppressor genes or a customized gene set for viewing the expression value. Users can also view the population level data to compare the patient expression value with the belonging cancer group through box plot. Users also can select from one of 24 cancer types to have an overview of this cancer studies. The summary view of the Summary page (Fig. 11b) provides users with a statistics view of the clinical attributes in and bar charts formats. Users can select a specific category such as female gender by click that component and only focus on that condition and other components will update the graphics accordingly such as the Patient List (Fig. 11c), FPKM and Feature Count chart on the Omics view (Fig. 11d). User can also view the individual patient information by click the entity in the patient list.

#### EndometriosisKB data

**Fig. 12:**
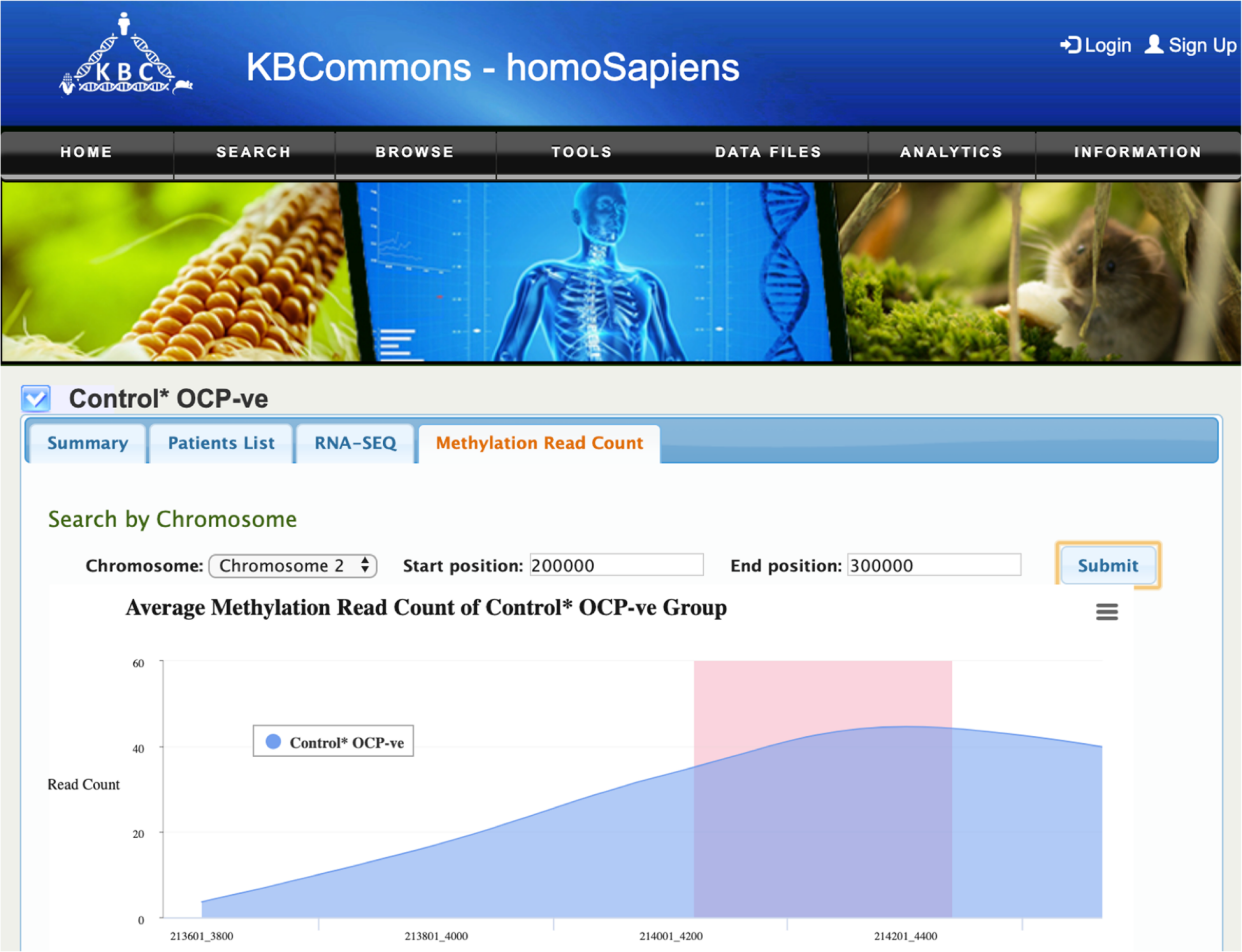
Example of Methylation Read Count page. The Methylation Read Count page visualizes Read Count with blue line and highlights significant CpG island regions in red

The Components of Endometriosis section can be viewed similarly to cancer data for datasets generated by Akter et [45]. The transcriptomics evidence shows RNAseq readcount and the epigenomics evidence including the methylation read count. The upstream and downstream region read count values of gene are added for methylation. Additionally, on the Methylation Read Count page (Fig. 12), the data are visualized by chromosome and region instead of gene via area-spline chart for methylation read count. Significant CpG island regions are highlighted in red.

### Download genome and OMICS data

KBCommons provides capacity for user to download the genome and multi-omics data based on their data accessibility level. Users can go to Download Data File tool and download any dataset they are interested in. User can choose the appropriate genome version first. Then choose the data type by selecting the primary data type in second selector and the subtype will be showing below accordingly. For experimental data, user also need to choose the data set if they have access for them. Finally, user needs to enter the gene/protein/metabolites set or chromosome coordinate to get the data downloaded.

### RNAseq analysis workflow

The RNAseq Analytics Workflow is intended for biological researchers, with no or limited computational or informatics expertise for their easy conduct. Creating a CyVerse [11, 12] account and enabling data in CyVerse data sharable are required. On the Create Workflow tab, users can upload Fastq read files contained multiple replicates and generated in two different experimental conditions. The appropriate gene annotation file and reference genome file is required to uploaded in format of GTF and Fasta respectively. In the Monitor Workflow tab, all of workflows user created with workflowID, time of creation and status of workflow are display in form of table. Users can retrieve workflow result by searching workflow ID on the Results page.

## Discussion

Many genome databases and multi-omics databases have been developed. For instance, MaizeGDB [1], Saccharomyces Genome Database [2], Ensembl genome browser [3] and Phytozome [4] are comprehensive integrated biological databases for vertebrate genomes and plant genomes. The Gene Expression Omnibus (GEO) [5] and the NCBI BioSystems database [6] are multi-omics database for analyzing gene profiling. Although these databases provide organism-specific genomic information and facilitate large-scale genomics studies, their multi-omics data are often individually scattered across different repositories making users hard to integrate them. KBCommons is an all-inclusive framework integrating plants, animals, microbes, viruses and biomedical diseases genome data and multi-omics dataset. It provides an array of tools for analyzing and visualization with multi-omics data. KBCommons allows users to create new databases and upload new multi-omics dataset without requiring users to reinvent the wheel and instead allowing them to focus more on their research by making such a centralized framework available for all organisms.

In the future, we will add more strategies to check and prevent improper, incorrect or false-positive data contribution to KBCommons. New functionalities will be added to KBCommons especially for enabling other tools such as protein-protein interaction viewer, eFP Browser [46], NGS Browser and SNPViz [47] tools. Many other new functionalities are currently under development. For example, we are developing Match Seq, a tool for aligning and visualizing ChIPSeq, DAPSeq, and RNASeq data, for patterns of gene expression discovery. In the subsequent versions, more advancements will be done for supporting multi-omics data integration and cross-species translational research automatically. We are also developing Restful APIs and FTP to access to datasets easily, and automated scripts to fetch publicly available multi-omics datasets from standard data sources such as GEO and NCBI BioSystems database.

## Conclusions

We have developed KBCommons, which provides a universal, comprehensive and one-stop shop framework for users to create new KBs, add more OMICs datasets and genome versions on their own and browse other information available in KBs. It also provides access to analytics services for various biological communities working on diverse organisms linked to CyVerse data store and XSEDE. It is implemented using HTML, JavaScript, Laravel PHP framework, and MySQL in web-based service and data storage for stable and fast access. It has data sharing capacities to control different levels of access from public to private. It provides a service that empowers users to create and contribute the genomics database, and that utilizes a suite of web-based tools to analyze, retrieve and visualize their data in addition to bringing in publicly available datasets. KBCommons facilitates a model where users can contribute and consume multi-omics datasets from their own and other labs in the same framework, with enhanced analytics and data access capabilities.

## Data Availability

All of the genome data and some of experimental data were collected from public data sources. The datasets generated and analyzed by our users during the current study are not publicly available due to private access.

## Abbreviations

CCLE: Cancer Cell Line Encyclopedia
HPC: High-Performance Computing
KEGG: Kyoto Encyclopedia of Genes and Genomes
NGS: Next-generation sequencing
SMILES: Simplified Molecular Input Line Entry Specification
SNP: single-nucleotide polymorphism
TCGA: The Cancer Genome Altas
XSEDE: The Extreme Science and Engineering Discovery Environment

## References

[1] Lawrence CJ, Dong Q, Polacco ML, Seigfried TE, Brendel V (2004). MaizeGDB, the community database for maize genetics and genomics. Nucleic Acids Res.

[2] Cherry JM, Hong EL, Amundsen C, Balakrishnan R, Binkley G, Chan ET, Christie KR, Costanzo MC, Dwight SS, Engel SR (2012). Saccharomyces genome database: the genomics resource of budding yeast. Nucleic Acids Res.

[3] Stalker J, Gibbins B, Meidl P, Smith J, Spooner W, Hotz H-R, Cox AV (2004). The Ensembl web site: mechanics of a genome browser. Genome Res.

[4] Rokhsar DS, Fazo J, Putnam N, Hayes RD, Neupane R, Howson R, Shu S, Mitros T, Hellsten U, Dirks W (2011). Phytozome: a comparative platform for green plant genomics. Nucleic Acids Res.

[5] Edgar R, Domrachev M, Lash AE (2002). Gene expression omnibus: NCBI gene expression and hybridization array data repository. Nucleic Acids Res.

[6] Geer LY, Marchler-Bauer A, Geer RC, Han L, He J, He S, Liu C, Shi W, Bryant SH (2010). The NCBI BioSystems database. Nucleic Acids Res.

[7] Joshi T, Fitzpatrick MR, Chen S, Liu Y, Zhang H, Endacott RZ, Gaudiello EC, Stacey G, Nguyen HT, Xu D (2014). Soybean knowledge base (SoyKB): a web resource for integration of soybean translational genomics and molecular breeding. Nucleic Acids Res.

[8] Joshi T, Patil K, Fitzpatrick MR, Franklin LD, Yao Q, Cook JR, Wang Z, Libault M, Brechenmacher L, Valliyodan B (2012). Soybean Knowledge Base (SoyKB): a web resource for soybean translational genomics. BMC Genomics.

[9] Zeng S, Narisetti SRK, Lyu Z, Joshi T (2017). KBCommons: A multi ‘OMICS’ integrative framework for database and informatics tools. 2017 IEEE International Conference on Bioinformatics and Biomedicine (BIBM): 13–16 Nov. 2017.

[10] Liu Y, Khan SM, Wang J, Rynge M, Zhang Y, Zeng S, Chen S, Maldonado dos Santos JV, Valliyodan B, Calyam PP (2016). PGen: large-scale genomic variations analysis workflow and browser in SoyKB. BMC Bioinformatics.

[11] Goff SA, Vaughn M, McKay S, Lyons E, Stapleton AE, Gessler D, Matasci N, Wang L, Hanlon M, Lenards A (2011). The iPlant collaborative: Cyberinfrastructure for plant biology. Front Plant Sci.

[12] Merchant N, Lyons E, Goff S, Vaughn M, Ware D, Micklos D, Antin P (2016). The iPlant collaborative: Cyberinfrastructure for enabling data to discovery for the life sciences. PLoS Biol.

[13] Towns J, Cockerill T, Dahan M, Foster I, Gaither K, Grimshaw A, Hazlewood V, Lathrop S, Lifka D, Peterson GD (2014). XSEDE: accelerating scientific discovery. Comput Sci Eng.

[14] Foundation AS (1999). Apache.

[15] Otwell T (2011). Laravel.

[16] Netscape Communications Corporation MF (1995). Ecma International: JavaScript.

[17] Angular JS (2010). Google.

[18] Mark Otto JT (2011). Bootstrap.

[19] Highsoft (2019). Highcharts.

[20] Google (2010). Google Charts.

[21] AB M (1995). MySQL.

[22] Inc. M (2009). MongoDB.

[23] Gv R (2010). Python 2.7.

[24] McKinney W (2008). Pandas: Python Data Analysis Library.

[25] Frankish A, Abdul Salam AI, Vullo A, Zadissa A, Winterbottom A, Parton A, Yates AD, Thormann A, Parker A, McMahon AC (2018). Ensembl 2019. Nucleic Acids Res.

[26] Trapnell C, Williams BA, Pertea G, Mortazavi A, Kwan G, van Baren MJ, Salzberg SL, Wold BJ, Pachter L (2010). Transcript assembly and quantification by RNA-Seq reveals unannotated transcripts and isoform switching during cell differentiation. Nat Biotechnol.

[27] Law CW, Chen Y, Shi W, Smyth GK (2014). Voom: precision weights unlock linear model analysis tools for RNA-seq read counts. Genome Biol.

[28] McCarthy DJ, Smyth GK, Robinson MD (2009). edgeR: a bioconductor package for differential expression analysis of digital gene expression data. Bioinformatics.

[29] Lerdorf R (2004). PHP 5.0.

[30] Barretina J, Caponigro G, Stransky N, Venkatesan K, Margolin AA, Kim S, Wilson CJ, Lehár J, Kryukov GV, Sonkin D (2012). The Cancer cell line encyclopedia enables predictive modelling of anticancer drug sensitivity. Nature.

[31] Tomczak K, Czerwińska P, Wiznerowicz M (2015). The Cancer genome atlas (TCGA): an immeasurable source of knowledge. Contemp Oncol.

[32] Kozomara A, Birgaoanu M, Griffiths-Jones S (2018). miRBase: from microRNA sequences to function. Nucleic Acids Res.

[33] Kanehisa M, Goto S (2000). KEGG: Kyoto encyclopedia of genes and genomes. Nucleic Acids Res.

[34] Finn RD, Coggill P, Eberhardt RY, Eddy SR, Mistry J, Mitchell AL, Potter SC, Punta M, Qureshi M, Sangrador-Vegas A (2016). The Pfam protein families database: towards a more sustainable future. Nucleic Acids Res.

[35] Thomas PD, Campbell MJ, Kejariwal A, Mi H, Karlak B, Daverman R, Diemer K, Muruganujan A, Narechania A (2003). PANTHER: a library of protein families and subfamilies indexed by function. Genome Res.

[36] Weininger D (1988). SMILES, a chemical language and information system. 1. Introduction to methodology and encoding rules. J Chem Inf Comput Sci.

[37] Langfelder P, Horvath S (2008). WGCNA: an R package for weighted correlation network analysis. BMC Bioinformatics.

[38] Thijs Gert, Marchal Kathleen, Lescot Magali, Rombauts Stephane, De Moor Bart, Rouzé Pierre, Moreau Yves (2002). A Gibbs Sampling Method to Detect Overrepresented Motifs in the Upstream Regions of Coexpressed Genes. Journal of Computational Biology.

[39] Thijs G, Moreau Y, De Smet F, Mathys J, Lescot M, Rombauts S, Rouze P, De Moor B, Marchal K (2002). INCLUSive: integrated clustering, upstream sequence retrieval and motif sampling. Bioinformatics.

[40] Altschul SF, Madden TL, Schäffer AA, Zhang J, Zhang Z, Miller W, Lipman DJ (1997). Gapped BLAST and PSI-BLAST: a new generation of protein database search programs. Nucleic Acids Res.

[41] Larkin MA, Blackshields G, Brown NP, Chenna R, McGettigan PA, McWilliam H, Valentin F, Wallace IM, Wilm A, Lopez R (2007). Clustal W and Clustal X version 2.0. Bioinformatics.

[42] Saitou N, Nei M (1987). The neighbor-joining method: a new method for reconstructing phylogenetic trees. Mol Biol Evol.

[43] Hibbert DB. Unweighted Pair Group Method With Arithmetic Mean (UPGMA). IUPAC Standards Online. 2017. 10.1515/iupac.88.0132.

[44] Plotly Technologies Inc: Collaborative data science. 2015.

[45] Akter S, Xu D, Nagel SC, Bromfield JJ, Pelch K, Wilshire GB, Joshi T (2019). Machine learning classifiers for endometriosis using Transcriptomics and Methylomics data. Front Genet.

[46] Winter D, Vinegar B, Nahal H, Ammar R, Wilson GV, Provart NJ (2007). An “electronic fluorescent pictograph” browser for exploring and analyzing large-scale biological data sets. PLoS One.

[47] Langewisch T, Zhang H, Vincent R, Joshi T, Xu D, Bilyeu K (2014). Major soybean maturity gene haplotypes revealed by SNPViz analysis of 72 sequenced soybean genomes. PLoS One.

